# Evaluation of Cardiomegaly in Dogs Using the Manubrium Heart Score Method and Determination of Its Diagnostic Accuracy in Comparison with the Vertebral Heart Score

**DOI:** 10.3390/vetsci12070619

**Published:** 2025-06-25

**Authors:** Bengü Bilgiç, Onur İskefli, Michela Pugliese, Mehmet Erman Or

**Affiliations:** 1 Department of Internal Medicine, Faculty of Veterinary Medicine, Istanbul University-Cerrahpasa, Istanbul 34320, Türkiye; ermanor@iuc.edu.tr; 2 Veterinary Cardiology Center Veterinary Clinic, Üsküdar, Istanbul 34660, Türkiye; onur.iskefli@istanbul.edu.tr; 3 Department of Veterinary Sciences, Faculty of Veterinary Medicine, University of Messina, 98168 Messina, Italy; michela.pugliese@unime.it

**Keywords:** cardiomegaly, manubrium, heart, dogs

## Abstract

Vertebral Heart Score (VHS) is one of the commonly used methods for detecting cardiomegaly in dogs. However, this method has diagnostic limitations due to factors such as breed variations, orthopedic disorders, anatomical anomalies, and operator-dependent subjectivity. The aim of this study was to evaluate the diagnostic utility of the Manubrium Heart Score (MHS) as an alternative to VHS in the assessment of cardiomegaly in dogs. A total of 490 dogs were classified and grouped based on body weight and cardiac health status. On the right lateral thoracic radiographs, MHS was calculated as the ratio of manubrium length (ML) to the sum of the long-axis heart length (cLAL) and short-axis heart length (cSAL). Similarly, VHS was determined. A positive correlation between VHS and MHS, as well as between ML and cSAL/cLAL, were observed in all groups except for the group of medium sized dogs with heart diseases. No correlations were found between MHS and LA or the LA/Ao ratio. In pairwise comparisons of VHS and MHS between heart-diseased and healthy dogs, the mean VHS showed a statistically significant difference in heart-diseased dogs (*p* < 0.001), and not across all groups (*p* > 0.05). MHS may not consider a useful method as an alternative to VHS.

## Simple Summary

Vertebral heart size (VHS) is widely used in clinical practice as a useful objective method to assess the dimensions of the cardiac silhouette. However, this method has several limitations related to breed variations, morphotype, orthopedic conditions, anatomical anomalies and operator-dependent factors. The aim of this study was to evaluate the diagnostic utility of the Manubrium Heart Score (MHS) as an alternative to VHS in the evaluation of cardiac enlargement in dogs. A total of 490 dogs were classified and grouped according to body weight and cardiac health status. VHS and MHS were calculated for each dog. A positive correlation between VHS and MHS was observed in all groups except for medium sized dogs with heart disease. No correlations were observed between MHS and left atrial dimension. Statistically significant differences were observed between VHS and MHS in dogs with heart disease (*p* < 0.001), but not in all groups (*p* > 0.05). MHS may not be a valuable tool to use as an alternative to VHS for cardiac enlargement.

## 1. Introduction

Acquired or congenital heart diseases are commonly observed in dogs and are characterized by various progressive symptoms that may lead to fatal outcomes [1,2,3]. In dogs with heart failure, the left ventricular remodeling process begins in the preclinical stage and reaches its peak in patients with congestive heart failure (CHF). Due to impaired cardiac activity, cardiac remodeling occurs, leading to alterations in myocardial size, shape, and function as a result of molecular, cellular, and interstitial changes [4]. Depending on the location and type of underlying lesions, cardiac chamber enlargement may be generalized, affecting all heart chambers, or may be confined to only the left or right side, or even a single chamber. The type of cardiac chamber enlargement typically develops as an adaptive response to chronic increases in cardiac volume load (e.g., valvular insufficiencies and left-to-right shunts) or increased systolic pressure (e.g., ventricular outflow obstruction or arterial hypertension). In cases of mitral or tricuspid insufficiency, the chambers on both sides of the affected valve gradually enlarge due to the regurgitant volume. The increase in volume and the associated cardiomegaly generally progress gradually unless an acute event, such as chordae tendineae rupture or the onset of atrial fibrillation, occurs [5]. Although echocardiography is considered the gold standard for the diagnosis of heart disease, thoracic radiography is one of the most commonly used diagnostic methods of assessing cardiac enlargement in dogs.

Radiographic evaluation allows for the non-invasive assessment of changes in heart size and shape, pulmonary vasculature, and congestion [6,7]. Several radiographic measurement methods such as computer-aided detection methods [8], the corrected heart volume index [9], the vertebral left atrial score (VLAS) [10,11], the thoracic inlet heart measurement [12,13], the thoracic vertebral length-to-height ratio [14] and the vertebral heart score (VHS) [15,16] have been proposed for evaluating cardiomegaly.

The majority of these methods are not widely used in clinical practice due to their limitations [17,18]. Among these, radiographic left atrial dimension (RLAD) measurement can be limited by difficulties in visualizing the left atrial border, especially in cases with overlapping pulmonary structures [19]. Similarly, VLAS requires precise identification of the carina and vertebral landmarks, which may not always be clearly visible depending on radiographic positioning or individual anatomical differences. Additional challenges common to all these techniques include misalignment of the cardiac silhouette, variations in thoracic conformation, differences in the respiratory phase during radiographic acquisition, challenges in achieving optimal patient positioning, and coexisting pulmonary conditions that can alter the cardiothoracic ratio. These factors can reduce the reliability and reproducibility of radiographic cardiac size assessments in some clinical cases [19,20].

The vertebral heart score (VHS) is a widely used method in contemporary cardiology practice for assessing cardiomegaly in dogs [15,21]. Various studies have proposed reference values for VHS in several healthy dog breeds [10,22,23,24,25,26,27,28,29,30,31,32]. However, certain factors may limit the diagnostic accuracy of VHS, particularly in chondrodystrophic breeds such as pugs, bulldogs, and Boston terriers. These limitations include common vertebral anomalies such as hemivertebrae, breed-specific variations in vertebral body length relative to overall body size, overestimation of the number of vertebrae, vertebral defects and deformities, as well as breed- and size-related measurement differences [18,21]. Consequently, as an alternative to VHS, the manubrium heart score (MHS) has been proposed for evaluating cardiomegaly in dogs [33]. The MHS method is similar to the VHS. It takes into account the measurements of the cardiac silhouette in the short and long-axis and compares them with the length of the sternal manubrium, existing a strong and still correspondence between the length of the manubrium and the corresponding cardiac axes [33]. Since it is highly distinct on lateral thoracic radiographs, regularly elongated, and easily identifiable in the shape of a bullet or a rectangular bone segment, MHS can be readily measured. Additionally, the calculation of cardiothoracic ratios may present challenges in clinical practice due to the need for specialized software and the marked variation in thoracic cavity conformation among different breeds. MHS measurements have been proposed as a viable alternative method that may overcome these difficulties [33,34]. Accurate assessment of cardiomegaly is of clinical importance, as it plays a key role in classification of myxomatous mitral valve disease, determining prognosis, guiding therapeutic decisions, and monitoring the progression of cardiac disease in patients [35]. Due to the various limitations outlined in the present study, the MHS method was evaluated as a potential reliable alternative to VHS. This study aims to determine the diagnostic value of MHS as an alternative to VHS for assessing cardiomegaly in different dog breeds and to evaluate its correlation with VHS.

## 2. Materials and Methods

### 2.1. Study Design

A total of 490 electronic medical records of dogs of different ages, breeds, weights and sexes referred to the Veterinary Hospital of Istanbul University-Cerrahpaşa, Faculty of Veterinary Medicine between 2020 and 2024 were reviewed with the approval of the Unit Ethics Committee of the Faculty of Veterinary Medicine, Istanbul University-Cerrahpaşa (Approval Report No: 2020/23) and in accordance with the national regulations outlined by the Turkish Ministry of Agriculture and Forestry (Regulation on the Welfare and Protection of Animals Used for Experimental and Other Scientific Purposes, Official Gazette No: 28141, dated 15 February 2012), which are in line with the European Union Directive 2010/63/EU on the protection of animals used for scientific purposes. Dogs that were obese or cachectic, dogs with a diagnosis of pericardial effusion, and dogs with poor quality radiographs that would make proper evaluation difficult were excluded from the study, as well as dogs presenting vertebral abnormalities such as scoliosis, kyphosis and hemivertebra or deformed manubria resulting from previous trauma detected at radiographic examination, in order to avoid inaccurate measurements.

Inclusion criteria were healthy dogs referred for cardiologic screening at the owner’s request or for a pre-anesthetic evaluation prior to standard surgical procedures (such as neutering or dental cleaning). Dogs were considered clinically healthy at the time of the complete cardiac examination if there was no murmur heard on auscultation and no evidence of morphological or hemodynamic cardiac abnormalities on echocardiographic and radiographic evaluation.

Based on the results of cardiological examinations, the dogs were categorized into study groups according to their health status and body weight as follows:

- A1: Dogs with heart disease, >2 kg and ≤10 kg
- B1: Dogs with heart disease, >10 kg and ≤20 kg
- C1: Dogs with heart disease, >20 kg and ≤40 kg
- A2: Healthy dogs, >2 kg and ≤10 kg
- B2: Healthy dogs, >10 kg and ≤20 kg
- C2: Healthy dogs, >20 kg and ≤40 kg

Considering that body weight in dogs can directly influence both cardiac size and thoracic conformation, the study population was divided into small (2–10 kg), medium (10–20 kg), and large (20–40 kg) breed groups. Given that radiographic measurements used to assess cardiac size, such as VHS and MHS, may vary according to body size, this classification aimed to facilitate the evaluation of size-related variations and to minimize potential bias or misleading results in the analysis.

The distribution of age, sex, and body weight (kg) of the dogs in each group is presented in Table 1.

A comprehensive cardiological examination was performed on all dogs. Cardiological evaluation encompassed echocardiographic, electrocardiographic (ECG), and radiographic assessments. All echocardiographic examinations and reporting were performed by a veterinary cardiologist throughout the duration of the study. Similarly, all radiographic measurements were conducted by a single evaluator to ensure consistency and minimize inter-observer variability. Echocardiographic evaluation was conducted using a Vetus 8 Doppler device (Mindray^®^, Shenzhen, China). The following M-mode measurements were recorded: IVSd (cm), LVIDd (cm), LVPWd (cm), IVSs (cm), LVIDs (cm), LVPWs (cm), EDV (ml), ESV (ml), EF (%), and FS (%). Additionally, the 2D measurements included LA (cm), Ao (cm), and LA/Ao, while Doppler assessments involved MVEmax (cm/s), MVAmax (cm/s), MVE/A, TVEmax (cm/s), TVAmax (cm/s), TVE/A, MRVmax (m/s), TRVmax (m/s), Avmax (cm/s), and Pvmax (cm/s). ECG evaluation was performed using CONTEC^®^ ECG600G model 6-channel ECG device (CONTEC^®^, Shijiazhuang, China), with the assessment of potential tachyarrhythmias, bradyarrhythmias, and conduction blocks in lead II.

**Figure 1:**
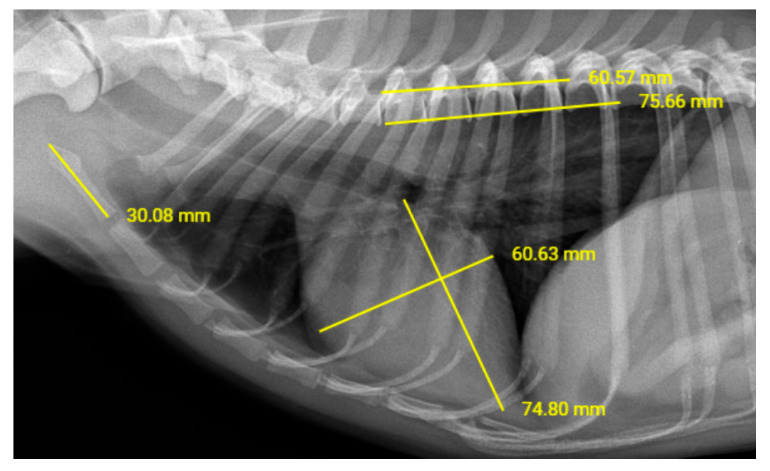
Measurements of ML and VHS in a Dog.

Radiographic examinations were conducted using the SMS CM-N model digital X-ray device (EcoRay^®^, Seoul, Republic of Korea). All radiographic procedures were performed according to a standardized protocol including control radiation exposure and source-to-image receptor distances. The dogs were restrained manually. Care was taken to avoid spinal or cardiac misalignment, which could lead to geometric distortions or make it difficult to visualize anatomical landmarks correctly. Radiographic images were digitalized and stored using an image archiving communication system and a proper diagnostic workstation. All thoracic radiographs included in the study were taken at the time of peak inspiration of the imaged dog, without use of sedation or anesthesia. VHS and MHS measurements were recorded from right lateral thoracic radiographs (Figure 1). VHS was determined by summing the long- and short-axis of the heart and measuring the number of vertebrae spanned from the cranial end of the fourth thoracic vertebra. MHS measurements were calculated using the following formula:

MHS = (cLAL + cSAL)/ML

(cLAL: Cardiac long-axis length, cSAL: Cardiac short-axis length, ML: Manubrium length).

### 2.2. Statistical Analysis

All statistical analyses were performed using SPSS package for Windows (version 25.0, IBM Corp., Armonk, NY, USA). A significance level of *p* < 0.05 was considered statistically significant. The Mann-Whitney U test was used for pairwise comparisons of MHS and VHS values between dogs with heart disease and healthy dogs, while Spearman correlation tests were applied to determine correlations between variables.

## 3. Results

The dog breeds included in each group were recorded as follows:

A1 group (*n* = 140): Cavalier King Charles (*n* = 30), Pomeranian (*n* = 18), Pekingese (*n* = 16), Maltese Terrier (*n* = 12), Miniature Pinscher (*n* = 12), mixed breeds (*n* = 11), Yorkshire Terrier (*n* = 9), Cocker Spaniel (*n* = 7), Toy Terrier (*n* = 6), Jack Russell Terrier (*n* = 5), Shih Tzu (*n* = 5), Pug (*n* = 3), Chihuahua (*n* = 2), Tibetan Spaniel (*n* = 2), Russian Toy (*n* = 1), and Spitz (*n* = 1).

A2 group (*n* = 100): Yorkshire Terrier (*n* = 13), Pomeranian (*n* = 12), Maltese Terrier (*n* = 12), mixed breed (*n* = 12), Pekingese (*n* = 8), Jack Russell Terrier (*n* = 8), Cavalier King Charles (*n* = 7), Miniature Pinscher (*n* = 7), Chihuahua (*n* = 5), Shih Tzu (*n* = 4), Pug (*n* = 3), Cocker Spaniel (*n* = 3), Poodle (*n* = 3), French Bulldog (*n* = 2), and Russian Toy (*n* = 1).

B1 group (*n* = 100): Cocker Spaniel (*n* = 34), Cavalier King Charles (*n* = 30), mixed breed (*n* = 15), Maltese Terrier (*n* = 6), Pekingese (*n* = 3), Jack Russell Terrier (*n* = 3), Beagle (*n* = 2), English Setter (*n* = 2), Russian Toy (*n* = 1), Bulldog (*n* = 1), Pug (*n* = 1), Husky (*n* = 1), and Golden Retriever (*n* = 1).

B2 group (*n* = 40): Cocker Spaniel (*n* = 12), mixed breed (*n* = 10), French Bulldog (*n* = 5), Cavalier King Charles (*n* = 2), Pug (*n* = 2), Russian Toy (*n* = 2), Golden Retriever (*n* = 2), Beagle (*n* = 2), Jack Russell Terrier (*n* = 1), Husky (*n* = 1), and Corgi (*n* = 1).

C1 group (*n* = 50): Golden Retriever (*n* = 27), mixed breed (*n* = 5), Labrador Retriever (*n* = 4), Cocker Spaniel (*n* = 4), German Shepherd (*n* = 2), Kangal (*n* = 2), Pitbull (*n* = 2), Mastiff (*n* = 1), Husky (*n* = 1), Great Dane (*n* = 1), and Poodle (*n* = 1).

C2 group (*n* = 60): Golden Retriever (*n* = 23), German Shepherd (*n* = 7), Labrador Retriever (*n* = 7), mixed breed (*n* = 6), Rottweiler (*n* = 4), Kangal (*n* = 3), Husky (*n* = 2), Dogo Argentino (*n* = 2), Pitbull (*n* = 2), English Setter (*n* = 1), American Staffordshire Terrier (*n* = 1), Pointer (*n* = 1), and Cane Corso (*n* = 1).

Dogs diagnosed with myxomatous mitral valve disease (A1, *n* = 112; B1, *n* = 81; C1, *n* = 15), tricuspid valve disease (A1, *n* = 12; B1, *n* = 7; C1, *n* = 2), hypertrophic cardiomyopathy (A1, *n* = 10, B1, *n* = 6; C1, *n* = 2), and dilated cardiomyopathy (A1, *n* = 6; B1, *n* = 6, C1, *n* = 31) based on cardiological examination were included in the study.

The echocardiographic findings of group A1, B1, and C1 are presented in Table 2 and A2, B2, and C2 in Table 3.

The results of VHS and MHS measurements obtained from radiographic evaluation for groups A, B, and C are presented in Table 4.

**Figure 2:**
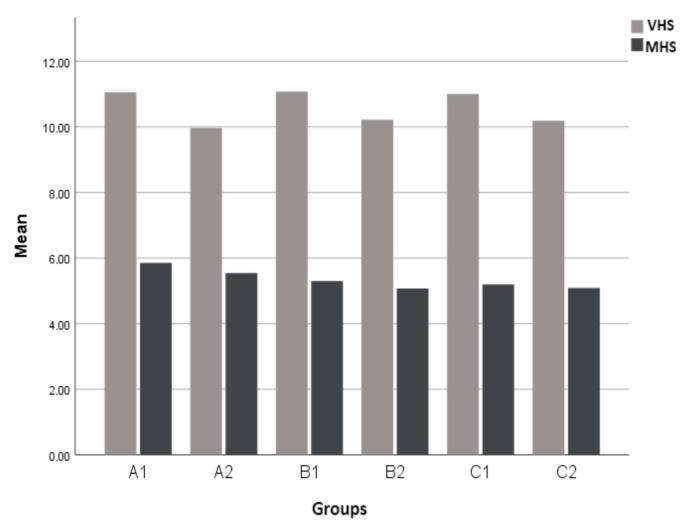
Bar chart comparing VHS and MHS data across A1, A2, B1, B2, C1, and C2 groups. The mean values are expressed in numbers of vertebral bodies for VHS and manubrium units (mu) for MHS.

As a result of the comparison of VHS and MHS values between healthy and heart-diseased dogs, a statistically significant difference was observed in mean VHS values between diseased and healthy dogs (*p* < 0.001). However, no statistically significant difference was observed in mean MHS measurements between diseased and healthy dogs (*p* > 0.05) (Table 5, Figure 2).

In the correlation analyses, a moderate positive correlation was observed between VHS and MHS in the A1 group (r = 0.41, *p* < 0.01). However, no correlation was found between MHS and LA or LA/Ao (r < 0.20, *p* > 0.05). A moderate positive correlation was detected between manubrium length and cLAL (r = 0.56, *p* < 0.01) as well as cSAL (r = 0.61, *p* < 0.01), while a weak positive correlation was found with LA/Ao (r = 0.19, *p* < 0.05). A moderate positive correlation was identified between VHS and MHS (r = 0.35, *p* < 0.01) in A2 group. However, no correlation was found between MHS and LA or LA/Ao (r < 0.20, *p* > 0.05). A moderate positive correlation was observed between manubrium length and both cLAL (r = 0.66, *p* < 0.01) and cSAL (r = 0.64, *p* < 0.01), whereas no correlation was detected between LA and LA/Ao (r < 0.20, *p* > 0.05).

In the B1 group, a moderate positive correlation was observed between VHS and MHS (r = 0.43, *p* < 0.01). However, no correlation was found between MHS and LA or LA/Ao (r < 0.20, *p* > 0.05). A moderate positive correlation was detected between manubrium length and both cLAL (r = 0.54, *p* < 0.01) and cSAL (r = 0.49, *p* < 0.01), whereas no correlation was observed between manubrium length and LA or LA/Ao (r < 0.20, *p* > 0.05). In the B2 group, no correlation was found between MHS and VHS, LA, or LA/Ao (r < 0.20, *p* > 0.05). However, a moderate positive correlation was observed between manubrium length and both cLAL (r = 0.68, *p* < 0.01) and cSAL (r = 0.69, *p* < 0.01), while no correlation was detected between LA and LA/Ao (r < 0.20, *p* > 0.05).

**Figure 3:**
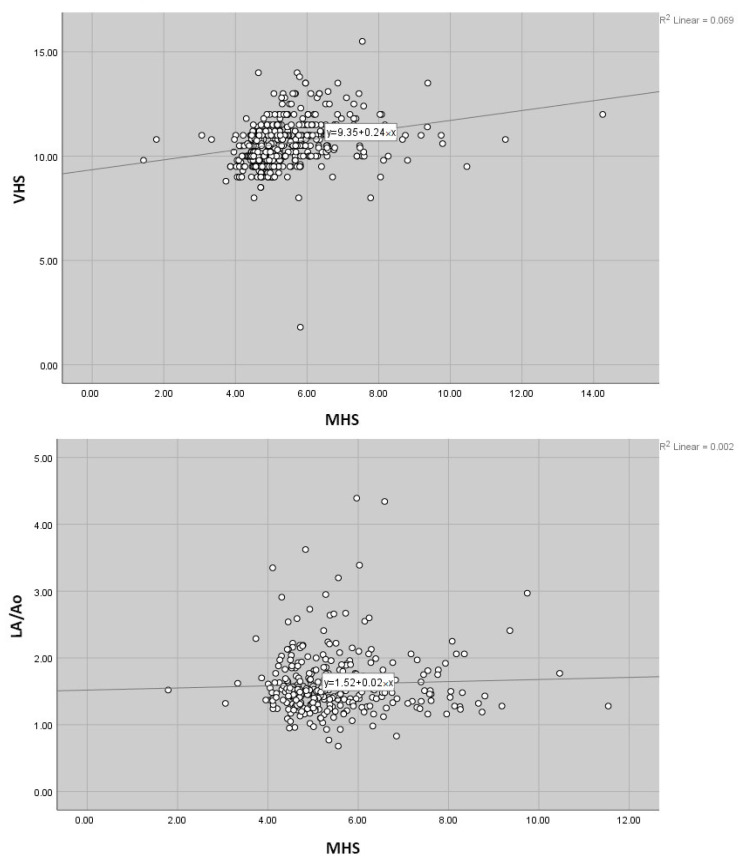
Simple scatter graphs show the correlations between MHS-VHS and MHS-LA/Ao.

In the C1 group, a moderate positive correlation was observed between VHS and MHS (r = 0.50, *p* < 0.01). However, no correlation was found between MHS and LA or LA/Ao (r < 0.20, *p* > 0.05). A moderate positive correlation was detected between manubrium length and both cLAL (r = 0.55, *p* < 0.01) and cSAL (r = 0.58, *p* < 0.01), whereas no correlation was observed between manubrium length and LA or LA/Ao (r < 0.20, *p* > 0.05). In the C2 group, a moderate positive correlation was found between VHS and MHS (r = 0.38, *p* < 0.01). However, no correlation was detected between MHS and LA or LA/Ao (r < 0.20, *p* > 0.05). A moderate positive correlation was observed between manubrium length and cLAL (r = 0.51, *p* < 0.01), while a weak positive correlation was found with cSAL (r = 0.32, *p* < 0.05). No correlation was observed between manubrium length and LA or LA/Ao (r < 0.20, *p* > 0.05) (Figure 3). Values between 0 and 0.3 (0 and −0.3) indicate a weak relationship; 0.3 and 0.7 (0.3 and −0.7) indicate a moderate relationship, and 0.7 and 1.0 (−0.7 and −1.0) indicate a strong relationship based on the criteria proposed by Ratner et al. (2009) [36].

In the multivariable regression analyses assessing the effects of age, body weight, and sex on ML, VHS, and MHS across the study groups, the A1 group showed a statistically significant positive association between body weight and ML (β = 0.185; *p* < 0.001). Within the same group, age was also positively associated with MHS (β = 0.120; *p* = 0.011). No significant effects of age, body weight, or sex were detected on VHS (*p* > 0.05). In the B1 group, none of the variables had a statistically significant effect on ML or MHS. However, male sex was found to have a significant negative effect on VHS (β = –0.482; *p* = 0.008). In the C1 group, both body weight (β = 0.140; *p* < 0.001) and male sex (β = 0.454; *p* = 0.005) were significantly associated with an increase in ML. In this group, none of the variables (age, weight, or sex) had a significant effect on MHS or VHS. In the A2 group, body weight significantly increased ML (β = 0.135; *p* < 0.001), and age was significantly associated with an increase in MHS (β = 0.133; *p* = 0.004). No significant effect was observed on VHS. In the B2 group, body weight had a statistically significant positive effect on ML (β = 0.144; *p* = 0.039), while all other associations were not statistically significant. In the C2 group, both body weight (β = 0.029; *p* = 0.002) and male sex (β = 0.721; *p* < 0.001) were significantly associated with an increase in ML. Age and weight did not have a significant effect on VHS.

## 4. Discussion

According to the consensus statement published by the American College of Veterinary Internal Medicine (ACVIM) in 2019, severe mitral regurgitation can initiate the process of cardiac remodeling through molecular, cellular, and structural changes that occur in the myocytes, interstitial tissue, and overall cardiac architecture of the left atrium and left ventricle. Although initially compensatory, this remodeling process may progressively impair cardiac function and ultimately result in volumetric enlargement of the entire heart or specific chambers, a condition defined as cardiomegaly. Efforts to identify reliable radiographic markers for cardiomegaly and cardiac enlargement are ongoing. However, definitive radiographic criteria for the clear identification of this stage have not yet been fully established. For this reason, a vertebral heart score (VHS) of ≥11.5 in general breeds, or a VHS value exceeding breed-specific reference ranges, has been proposed as a radiographic indicator of cardiomegaly [35]. From an echocardiographic perspective, the LA/Ao ratio has been shown to be an effective parameter for assessing left atrial enlargement in dogs [37]. In the present study, dogs with an LA/Ao ratio ≥1.6—measured in the right parasternal short-axis view at early diastole—were considered to have a potential risk of cardiomegaly.

Studies evaluating the relationship between cardiomegaly and MHS are quite limited. In this study, VHS and MHS values were comparatively evaluated in both healthy and heart-diseased dogs to assess the diagnostic role of MHS in detecting cardiomegaly. The evaluation was conducted separately for different dog breeds according to body weight. Since heart size varies depending on breed and body size, breed-specific VHS and MHS reference values have been established [15,31,32]. Therefore, to minimize variations related to breed size, study groups were categorized based on body weight. Challenges in measuring VHS in dogs with various acquired or congenital vertebral abnormalities, particularly in brachycephalic breeds [23], along with differences in breed-specific VHS [21,22,23,24,25,26,27,28,29,30,31,32] and operator-dependent subjectivity, have led to the exploration of alternative measurement methods. MHS was first introduced in 2017 by determining the ratio of the heart’s short-axis and long-axis lengths to the corresponding manubrium length in thoracic radiographic images of dogs, and its correlation with VHS was evaluated.

A previous similar study [31] reported that heart size measurements obtained from right lateral and ventrodorsal thoracic radiographic images of a total of 120 clinically healthy small and large breed dogs showed a strong correlation with manubrium length but a weak correlation with VHS in both small and large breed dogs. In the same study, a weak correlation between VHS and MHS was observed in large breed dogs, while no correlation was detected in small breed dogs. In our study, measurements obtained from right lateral thoracic radiographs of small, medium, and large breed dogs revealed a moderate correlation between both the long-axis and short-axis heart dimensions and manubrium length. This finding suggests that heart size and manubrium length exhibit a parallel relationship depending on breed size. However, unlike the previously reported study, our results demonstrated a moderate positive correlation between VHS and MHS in both healthy and heart-diseased small and large breed dogs, whereas no correlation was observed in healthy medium-sized dogs. The correlation detected between VHS and MHS in both healthy and heart-diseased dogs significantly weakens the diagnostic role of MHS in the assessment of cardiomegaly. However, as suggested by Mostafa and Berry (2017), the findings of our study also indicate that ML can serve as a suitable reference value for evaluating cSAL and cLAL in dogs [33]. However, cSAL and cLAL parameters are not commonly used as standalone criteria in the assessment of cardiomegaly; moreover, they vary depending on breed and body size in dogs. Both manubrium length and the axial dimensions of the heart (cSAL and cLAL) are positively associated with body size. In large breed dogs, both sternal length and cardiac dimensions naturally tend to be greater. Therefore, the positive correlation observed between ML and cSAL/cLAL most likely reflects proportional anatomical scaling relative to overall body size. In a different study [34], MHS was reported to provide useful and objective values that could aid in assessing potential heart diseases in dogs. Therefore, it was recommended that MHS be incorporated into the diagnostic tools used by veterinary practitioners for screening cardiac diseases in dogs. However, in our study, pairwise comparisons between healthy and heart-diseased dogs across all small, medium, and large breed groups revealed that while VHS values were statistically higher in dogs with heart disease compared to healthy dogs, MHS values did not show significant differences between healthy and diseased groups. By utilizing more consistent anatomical landmarks such as vertebral bodies, VHS is considered to be more standardized and sensitive. In contrast, MHS is more susceptible to variations in thoracic conformation and commonly encountered differences in sternum morphology, which may limit its sensitivity compared to VHS. Furthermore, no correlation was observed between MHS and echocardiographically measured left atrial diameter or LA/Ao ratio in all groups.

It should be noted that among the heart disease cases included in this study, some dogs were diagnosed with HCM, a condition in which cardiac remodeling primarily manifests as left ventricular wall hypertrophy rather than left atrial enlargement. As such, LA/Ao ratio may remain within normal limits in certain HCM cases. Therefore, relying solely on LA/Ao as an indicator of cardiomegaly may be insufficient in accurately reflecting cardiac enlargement in dogs with HCM. This limitation should be considered when interpreting LA/Ao values as part of the cardiomegaly assessment in this subgroup.

In both A1 and A2 groups, the absence of a correlation between VHS and MHS indicates that there is also no relationship between manubrium length and the number of vertebrae corresponding to the short- and long-axis dimensions of the heart, starting from the fourth thoracic vertebra. However, the lack of an association between MHS and LA or LA/Ao suggests that MHS cannot be used as a diagnostic tool for cardiomegaly caused by left atrial enlargement. Nevertheless, the positive correlation between manubrium length and the long- and short-axis dimensions of the heart reflects that manubrium length may be related to both the width and length of the heart and, consequently, to the overall body size of the dog. Similarly, although there is a weak correlation with LA/Ao in the A1 group, considering that manubrium length remains constant in adult dogs, it can be concluded that it does not have clinical diagnostic significance in assessing left atrial enlargement. Similar findings obtained in Group A were also valid for Group B, except for the MHS-VHS relationship in the B2 subgroup. The lack of a correlation between MHS and VHS in the B2 subgroup suggests that, in some healthy dog breeds within the 10–20 kg range, there is no relationship between manubrium length and the number of vertebrae corresponding to the short- and long-axis dimensions of the heart, starting from the fourth thoracic vertebra. This may be attributed to individual-specific variations in the length of thoracic vertebrae or the manubrium. The observation of similar findings in both healthy and heart-diseased dogs in groups A and C indicates that the relationship between cardiac lengths and manubrium lengths is similar in both small and large breed dogs. Although a positive correlation between VHS and MHS was found across all study groups, the MHS value did not show a statistically significant difference when comparing healthy and cardiac-diseased dogs. In contrast, the significant difference observed in VHS comparisons within the same groups supports the diagnostic reliability of VHS in the assessment of cardiomegaly [38,39].

In the study, the effects of age, body weight, and sex on ML, VHS, and MHS were also evaluated. Accordingly, body weight emerged as a consistent and positive determinant of ML. The influence of age on the ML, VHS and MHS was generally limited, with a significant increase in MHS observed only in the younger age groups (A1 and A2). The effect of sex varied depending on the group and the outcome; male sex was associated with increased ML in groups C1 and C2.

In addition to the known limitations of the VHS method—such as variability due to breed differences, orthopedic conditions, and anatomical anomalies—our study findings revealed that the length and shape of the manubrium may also vary considerably among breeds. Specifically, in brachycephalic breeds, which are genetically predisposed to vertebral anomalies and tend to have a more compact thoracic conformation, the manubrium was often shorter and irregular in shape. In contrast, dolichocephalic breeds, which possess a deeper and longer thoracic structure, generally exhibited a more prominent and elongated manubrium. Furthermore, in young dogs, the manubrium may not yet be fully ossified, appearing shorter and more rounded on radiographs. In cases where the manubrium is excessively short, malformed, or degenerated, the use of the MHS may yield misleading results. Based on these breed-related and individual variations observed during our study, it is evident that the manubrium length does not constitute a standardized reference point for all individuals.

Although the groups in our study were designed to include small, medium, and large breed dogs, the inclusion of dogs from different breeds, as well as variations in age and sex, represent limitations of the study.

## 5. Conclusions

MHS is not a useful method for assessing cardiomegaly in small, medium, and large breed dogs and that it is not suitable as an alternative to VHS. However, it was observed that manubrium length is correlated with both the short-axis and long-axis dimensions of the heart.

## Figures and Tables

**Table 1:** Distribution of age (years), weight (kg), and sex.

Study Groups	Age (Years) Mean (Min–Max)	Weight (kg) Mean (Min–Max)	Sex	
			Male	Female
A1 (*n* =140)	8.95 (2–17)	6.61 (2–10)	72	68
B1 (*n* = 100)	9.66 (2–18)	13.1 (10.2–19)	55	45
C1 (*n* = 50)	10.46 (3–14)	31.53 (25.5–45)	31	19
A2 (*n* = 100)	6.67 (1–17)	5.57 (1.20–9.5)	50	50
B2 (*n* = 40)	7.41 (1–16)	14.87 (10.5–20)	15	25
C2 (*n* = 60)	8.48 (1–15)	33.50 (20.5–53)	36	24

**Table 2:** Echocardiographic M-mode, 2D and Doppler measurements of A1, B1, and C1 groups.

	A1 (*n* = 140)		B1 (*n* = 100)		C1 (*n* = 50)	
M-Mode	Mean ± SD	Min–Max	Mean ± SD	Min–Max	Mean ± SD	Min–Max
IVSd (cm)	0.75 ± 0.18	0.33–1.34	0.89 ± 0.3	0.5–2.51	1.1 ± 0.29	0.5–1.8
LVIDd (cm)	2.75 ± 0.88	0.94–5.24	3.31 ± 0.72	1.22–4.92	4.4 ± 1.04	2.32–7.83
LVPWd (cm)	0.8 ± 0.87	0.06–9.67	0.84 ± 0.18	0.39–1.5	1.1 ± 0.28	0.67–1.86
IVSs (cm)	1.06 ± 0.28	0.54–1.97	1.19 ± 0.28	0.63–2.14	1.41 ± 0.29	0.75–2.22
LVIDs (cm)	1.47 ± 0.59	0.29–4.25	1.83 ± 0.49	0.5–3.04	2.62 ± 0.82	1.07–5.24
LVPWs (cm)	1.03 ± 0.22	0.72–1.97	1.2 ± 0.25	0.62–1.68	1.41 ± 0.3	0.81–2.2
EDV (mL)	29.99 ± 25.21	0.83–137.5	46.14 ± 25.04	3.5–119.1	97.26 ± 65.52	18.56–328.46
ESV (mL)	6.6 ± 9.1	0.06–80.68	10.26 ± 7.32	0.3–36.2	29.3 ± 26.55	3.93–144.04
EF (%)	81.09 ± 9.91	38.89–98.58	78.33 ± 10.36	40.85–96.3	72.13 ± 10.95	30.07–86.36
FS (%)	47.3 ± 9.95	19–78.38	44.23 ± 10.68	4.76–66.67	39.48 ± 8.69	13.64–53.57
2D	Mean ± SD	Min–Max	Mean ± SD	Min–Max	Mean ± SD	Min–Max
LA (cm)	2.12 ± 0.89	0.99–7.13	2.39 ± 0.564	1.29–4.35	2.96 ± 0.67	1.52–5.52
Ao (cm)	1.19 ± 0.33	0.6–2.61	1.41 ± 0.25	0.88–2.32	1.92 ± 0.37	1.16–2.61
LA/Ao	1.88 ± 0.65	0.83–4.39	1.73 ± 0.408	0.93–3.47	1.57 ± 0.33	0.77–2.21
Doppler	Mean ± SD	Min–Max	Mean ± SD	Min–Max	Mean ± SD	Min–Max
MVEmax (cm/s)	72.31 ± 31.38	26.93–200.14	73.71 ± 23.98	32.51–150.52	61.59 ± 15.27	36.2–119.53
MVAmax (cm/s)	60.26 ± 22.44	21.86–161.21	59.6 ± 18.33	23.97–104.45	57.6 ± 16.4	31.58–111.88
MVE/A	1.26 ± 0.46	0.41–2.64	1.3 ± 0.42	0.62–2.68	1.12 ± 0.29	0.48–1.92
TVEmax (cm/s)	64.03 ± 22.97	17.76–120.68	60.42 ± 15.69	28.53–126.61	59.81 ± 14.95	37.22–89.34
TVAmax (cm/s)	50.2 ± 17.59	19.66–105.60	48.64 ± 14.08	20.92–86.18	51.62 ± 13.29	31.7–81.2
TVE/A	1.31 ± 0.37	0.69–2.53	1.29 ± 0.32	0.75–2.29	1.2 ± 0.29	0.72–1.74
MRVmax (m/s)	4.48 ± 1.66	1.21–7.09	4.7 ± 1.43	1.12–6.78	4.25 ± 1.17	2.47–6.36
TRVmax (m/s)	2.77 ± 0.84	0.93–4.30	2.58 ± 0.54	1.58–3.38	2.29 ± 0.24	1.95–2.45
Avmax (cm/s)	98.26 ± 26.58	42–178	108.42 ± 27.54	58–224	123.23 ± 54.2	75–364
Pvmax (cm/s)	83.96 ± 25.57	30.68–204	94.11 ± 24.94	49–189	110.19 ± 70.95	56–505

IVSd: Interventricular septal diameter end diastole, LVIDd: Left ventricular internal diameter end diastole, LVPWd: Left ventricular posterior wall thickness in diastole, IVSs: Interventricular septal diameter in systole, LVIDs: Left ventricular internal diameter in systole, LVPWs: Left ventricular posterior wall thickness in systole, EDV: End-diastolic volume, ESV: End-systolic volume, EF: Ejection fraction, FS: Fractional shortening, LA: Left Atrium diameter, Ao: Aortic root diameter, LA/Ao: Ratio of Left Atrium to Aortic root diameter, MVEmax: Peak velocity of mitral valve E-wave, MVAmax: Peak velocity of mitral valve A-wave, MVE/A: Ratio of mitral valve E-wave to A-wave velocity, TVEmax: Peak velocity of tricuspid valve E-wave, TVAmax: Peak velocity of tricuspid valve A-wave, TVE/A: Ratio of tricuspid valve E-wave to A-wave velocity, MRVmax: Maximum velocity of mitral regurgitation, TRVmax: Maximum velocity of tricuspid regurgitation, Avmax: Maximum velocity of aortic valve flow, Pvmax: Maximum velocity of pulmonary valve flow.

**Table 3:** Echocardiographic M-mode, 2D and Doppler measurements of A2, B2, and C2 groups.

	A2 (*n* = 100)		B2 (*n* = 40)		C2 (*n* = 60)	
M-Mode	Mean ± SD	Min–Max	Mean ± SD	Min–Max	Mean ± SD	Min–Max
IVSd (cm)	0.7 ± 0.17	0.35–1.41	0.97 ± 0.24	0.67–1.64	1.17 ± 0.36	0.51–2.55
LVIDd (cm)	2.27 ± 0.47	1.23–4.16	3.07 ± 0.57	1.31–4.16	3.97 ± 0.67	1.45–5.69
LVPWd (cm)	0.68 ± 0.22	0.41–1.9	0.91 ± 0.15	0.57–1.17	1.13 ± 0.24	0.45–1.74
IVSs (cm)	0.93 ± 0.18	0.47–1.7	1.23 ± 0.25	0.79–1.77	1.47 ± 0.34	0.72–2.49
LVIDs (cm)	1.26 ± 0.37	0.39–2.31	1.7 ± 0.4	0.45–2.31	2.17 ± 0.59	0.59–3.63
LVPWs (cm)	0.93 ± 0.2	0.54–1.85	1.28 ± 0.18	0.94–1.63	1.51 ± 0.31	0.94–2.42
EDV (mL)	17.48 ± 10.57	2.57–76.71	36.85 ± 16.47	4.25–76.71	70.52 ± 27.18	5.53–159.58
ESV (mL)	4.11 ± 3.46	0.15–18.44	8.25 ± 4.92	0.22–18.44	16.31 ± 10.64	0.47–55.5
EF (%)	79.24 ± 9.97	38.89–96.57	78.27 ± 9.69	50.94–94.75	78.45 ± 8.61	62.5–97.12
FS (%)	45.78 ± 9.33	17.54–70.97	45.11 ± 8.78	25–65.71	46.08 ± 9.05	33.33–74.67
2D	Mean ± SD	Min–Max	Mean ± SD	Min–Max	Mean ± SD	Min–Max
LA (cm)	1.56 ± 0.37	0.69–2.76	2.03 ± 0.29	1.57–2.91	2.75 ± 0.54	1.5–4.44
Ao (cm)	1.12 ± 0.26	0.46–1.94	1.45 ± 0.22	1.12–1.84	1.96 ± 0.41	1–3.27
LA/Ao	1.4 ± 0.18	0.68–1.52	1.42 ± 0.16	0.97–1.56	1.41 ± 0.18	0.95–1.63
Doppler	Mean ± SD	Min–Max	Mean ± SD	Min–Max	Mean ± SD	Min–Max
MVEmax (cm/s)	59.98 ± 14.50	34.4–93.99	70 ± 17.96	41.34–108.43	69.4 ± 15	36.5–106.8
MVAmax (cm/s)	49.73 ± 17.96	1.68–127.8	57.82 ± 16.28	31.54–97.08	55.3 ± 14.1	30.6–91.6
MVE/A	1.28 ± 0.43	0.64–2.64	1.25 ± 0.29	0.8–1.99	1.3 ± 0.3	0.4–2.1
TVEmax (cm/s)	54.95 ± 16.85	0.67–103.29	59.84 ± 15.04	34.78–90.34	63.6 ± 16	37.7–126.8
TVAmax (cm/s)	48.78 ± 13.46	15.02–88.67	50.85 ± 14.46	24.7–78.67	52.2 ± 17.7	27–101.6
TVE/A	1.21 ± 0.34	0.65–2.26	1.22 ± 0.28	0.73–1.7	1.3 ± 0.3	0.8–2.3
Avmax (cm/s)	94.1 ± 24.79	48–161	122.07 ± 28.4	69–180	116.6 ± 28.4	69–179
Pvmax (cm/s)	85.6 ± 20.21	42–126	104.65 ± 24.22	68–165	101.5 ± 24.8	45–153

IVSd: Interventricular septal diameter end diastole, LVIDd: Left ventricular internal diameter end diastole, LVPWd: Left ventricular posterior wall thickness in diastole, IVSs: Interventricular septal diameter in systole, LVIDs: Left ventricular internal diameter in systole, LVPWs: Left ventricular posterior wall thickness in systole, EDV: End-diastolic volume, ESV: End-systolic volume, EF: Ejection fraction, FS: Fractional shortening, LA: Left Atrium diameter, Ao: Aortic root diameter, LA/Ao: Ratio of Left Atrium to Aortic root diameter, MVEmax: Peak velocity of mitral valve E-wave, MVAmax: Peak velocity of mitral valve A-wave, MVE/A: Ratio of mitral valve E-wave to A-wave velocity, TVEmax: Peak velocity of tricuspid valve E-wave, TVAmax: Peak velocity of tricuspid valve A-wave, TVE/A: Ratio of tricuspid valve E-wave to A-wave velocity, MRVmax: Maximum velocity of mitral regurgitation, TRVmax: Maximum velocity of tricuspid regurgitation, Avmax: Maximum velocity of aortic valve flow, Pvmax: Maximum velocity of pulmonary valve flow.

**Table 4:** Radiographic measurements in study groups.

	cLAL (cm)		cSAL (cm)		Manubrium (cm)		VHS (cm)		MHS (cm)	
Groups	Mean (Min–Max)	SD	Mean (Min–Max)	SD	Mean (Min–Max)	SD	Mean (Min–Max)	SD	Mean (Min–Max)	SD
A1 (*n* = 140)	8.17 (4.85–13.76)	1.62	6.91 (4.09–13.24)	1.43	2.68 (1.15–4.58)	0.73	11.05 (8.5–15.50)	1.12	5.85 (3.06–10.46)	1.38
A2 (*n* = 100)	7.30 (4.75–9.84)	1.09	6.02 (3.83–8.91)	0.99	2.54 (1.18–6.23)	0.75	9.96 (8–11.50)	0.61	5.54 (1.43–11.54)	1.28
B1 (*n* = 100)	10.15 (6.95–16.03)	1.50	8.36 (5.36–12.83)	1.16	3.57 (1.69–5.01)	0.59	11.07 (9–13.80)	0.91	5.29 (3.97–14.25)	1.17
B2 (*n* = 40)	10.09 (7.89–14.07)	1.29	8.11 (6.84–10.78)	0.84	3.61 (2.29–4.64)	0.44	10.21 (8.50–14)	0.89	5.07 (4.12–6.59)	0.56
C1 (*n* = 50)	14.02 (8.41–21.57)	2.18	11.38 (6.91–17.54)	1.80	4.94 (2.60–6.98)	0.82	11 (9.50–13.00)	0.68	5.19 (4.23–8.17)	0.69
C2 (*n* = 60)	13.59 (10.04–17.15)	1.35	10.82 (8.75–13.54)	1.08	4.91 (2.64–6.64)	0.79	10.18 (9–11.20)	0.59	5.08 (3.96–8.66)	0.90

cLAL: Cardiac long-axis length, cSAL: Cardiac short-axis length, VHS: Vertebral heart score, MHS: Manubrium heart score, SD: Standard deviation.

**Table 5:** Comparison of VHS and MHS values between healthy and heart disease groups.

A (2–10 kg)		Mean (Min–Max)	Median	SD	*p*
VHS	HD	11.05 (8.5–15.50)	11	1.12	*<0.001*
	Healthy	9.96 (8–11.50)	10	0.61	
MHS	HD	5.85 (3.06–10.46)	5.60	1.38	*0.071*
	Healthy	5.54 (1.43–11.54)	5.23	1.28	
B (10–20 kg)		Mean (Min–Max)	Median	SD	*p*
VHS	HD	11.07 (9–13.80)	11	0.91	*<0.001*
	Healthy	10.21 (8.50–14)	10.20	0.89	
MHS	HD	5.29 (3.97–14.25)	5.06	1.17	*0.527*
	Healthy	5.07 (4.12–6.59)	4.99	0.56	
C (20–40 kg)		Mean (Min–Max)	Median	SD	*p*
VHS	HD	11 (9.50–13.00)	11	0.68	*<0.001*
	Healthy	10.18 (9–11.20)	10	0.59	
MHS	HD	5.19 (4.23–8.17)	5.11	0.69	*0.065*
	Healthy	5.08 (3.96–8.66)	4.82	0.90	

VHS: Vertebral heart scale, MHS: Manubrium heart scale, HD: Heart disease, SD: Standard deviation.

## Data Availability

The data supporting the conclusions of this article will be made available by the authors on reasonable request.
